# Cell-Based Therapies for Myocardial Regeneration in Heart Failure: 20 Years of Debate

**DOI:** 10.21470/1678-9741-2020-0362

**Published:** 2020

**Authors:** Paulo André Bispo Machado-Júnior, Gustavo Gavazzoni Blume, Julio César Francisco, Luiz César Guarita-Souza

**Affiliations:** 1 Post-Graduation program in Health Sciences, Pontifícia Universidade Católica do Paraná (PUCPR), Curitiba, PR, Brazil.; 2 Universidade Positivo (UP), Curitiba, PR, Brazil.

Heart failure (HF) is still a challenging disease around the world, and despite the advances in pharmacological approaches and surgical techniques over the past few years, the recovery of the contractility of cardiomyocytes after an injury in the heart is still not feasible.

Heart transplantation remains as the most effective treatment for this patient population, and the techniques regarding this procedure had made huge progress since the first transplant, performed in 1967 by Dr Christiaan Barnard. Despite this, the challenges in candidate selection, shortage of organ donors, and comorbidities that may contraindicate the postoperative immunosuppression are factors that still limit the transplant to a selected group of patients. This scenario has encouraged research for alternative therapeutic approaches for HF, aiming the regeneration of the heart tissue^[1]^.

Several agents have been studied for this purpose: fetal cardiomyocytes, skeletal myoblasts, embryonic and adult stem cells (SC), synthetic polymers, human and non-human collagen gels, and decellularized tissues. These therapies aim to provide a cell-matrix integration that provides an improvement of global cardiac function either through differentiation into cardiomyocytes, paracrine, and immunomodulatory actions, or providing a three-dimensional structure that enables cell adhesion, survival, and proliferation.

Fetal cardiomyocytes were the agents initially evaluated, and the results showed capacity of colonization of the infarcted area with gain of cellular function. However, its use was abandoned due to the need for immunosuppression and ethical issues involved. Later, skeletal myoblasts were tested, principally for their self-regenerating characteristics, with regional functional improvements and colonization of the transplanted region; yet, by their non-differentiation into cardiomyocytes and the maintenance of their phenotypic characteristics, a process of fatty degeneration was commonly trigged, with consequent ventricular arrhythmias after the implantation, and the anti-remodeling effect in transmural infarction models was not observed^[2]^.

To date, the most studied agents are SC. These cells had raised great expectation on the part of researchers and patients about the differentiation potentials into other subtypes lines such as nervous, blood, bone, and cardiac tissue, due to the capacity of self-renewal, proliferation, and differentiation into other cell lines (Table 1).

**Table 1:** Comparison of the different cell-based approaches for myocardial regeneration in heart failure.

	Subtype	Origin	Benefits	Limitations
Stem cells	Embryonic stem cells (ESC)	Derived from the inner layer of the blastocyst	Potential of differentiation into the three embryonic layers: ectoderm, mesoderm, and endoderm	Ethical issues, immunological consequences, and malignancy potential
	Induced pluripotent stem cells (iPSC)	Mature somatic cells modified through induction of specific transcription factors in vitro	Similar to ESC, with the advantage of being easily obtained in vitro with great potential for expansion	Genomic instability during the culture process and teratogenic potentials
	Adult stem cells	Bone marrow, adipose tissue, muscle, umbilical cord, dental pulp, amniotic fluid	Diversity of cells, easy obtention, immunotolerance, and few ethical issues	Restricted ability of differentiation into other tissues
	**Differentiation capacity**			
	Totipotent	Any cell type in the body, including embryonic attachments		
	Pluripotent	Any cell type in the body, except for embryonic attachments		
	Multipotent	Only a few cell lines, with reduced potential for differentiation		
	**Biological properties**	**Mechanism of action**	**Benefits**	**Limitations**
**Human amniotic membrane**	Source of stem cells, anti-fibrotic/pro-angiogenic capacity, mechanical strength	Cardiac differentiation, immunomodulatory effects, and cell-matrix integration (scaffold)	Low immunogenicity, easily obtained and low cost	Only a few numbers of studies exploring the regeneration potential on the myocardium
**Intestinal submucosa**	Three-dimensional architecture, rich vascularization, and source of growth factors	Cell-matrix adhesion and integration	Easily obtained and low cost	Possible immunogenic consequences and few studies exploring its potential
**Skeletal myoblasts**	Self-regenerating characteristics	Myocardium colonization and cardiac contraction	Easily obtained, with significant improvements in cardiac function	Fatty degeneration process with subsequent arrythmias
**Hydrogels**	Biocompatibility, mechanical properties, and immunomodulatory capacity	Reduction of inflammatory response, angiogenesis, and cell proliferation	Easy application and low immunogenicity	High cost and low retention after cardiac injection

In 2001, Orlic et al.^[3]^ suggested the SC ability to transdifferentiate into cardiomyocytes after using bone marrow SC in a model of coronary artery ligation in rats, with significant improvements in ejection fraction on the animal model. These results were not replicated in subsequent experiments, but studies with SC advanced through several clinical trials around the world.

Due to the pluripotential characteristic and medium-dependent differentiation, the results of the studies were mainly related to the underlying pathologies in topic. In transmural infarction models, the functional benefit was discreet since these cells presented difficulty in differentiating into cardiomyocytes, similarly as in models of dilated cardiomyopathy with the predominance of fibrosis. In contrast, when the studies were related to ischemic pathologies, the results were more encouraging, justified by the possibility of neovascularization in the regions on which the cells were injected^[4]^.

Other studies evaluated the association of skeletal myoblasts and adult SC (cell coculture), based on the hypothesis that adult SC could stimulate neoangiogenesis and the myoblasts would provide the regeneration of the cell tissue, both in autologous form. The functional benefits were identified in both models of transmural infarction and Chagasic cardiomyopathy, but the left ventricular reverse remodeling effect was identified only in the Chagas model^[5]^. secretion of growth factors that are able to stimulate cell survival and

In this sense, it is not a current consensus status that the proliferation, and the production of angiogenic factors can stimulate mechanism of action of SC occurs through the differentiation the formation of new cardiac vessels in the ischemic area, while the into cardiomyocytes; other mechanisms such as paracrine and secretion of prostaglandin E2, nitric oxide, and transforming growth immunomodulatory effects had been more accepted recently. The factor-β is able to ameliorate the pro-inflammatory response^[6]^.

An important factor in determining the efficiency of the SC is related to its delivery method to the myocardium. The intravenous method is the easiest form of delivery, on the account of the simplicity of the method and the low invasiveness, allowing multiple and intermittent injections; despite this, studies had shown about 0% of cell retention in the myocardium, mainly related to the systemic circulation, which allows the cells to be deposited in other organs such as the lung and spleen.

The intracoronary route has the advantage of delivering the cells into a specific area of arterial irrigation, but this method does not allow the SC to reach non-perfused areas, decreasing its survival and replication. Another possibility is the intramyocardial injection, probably the most effective, since it provides a precise delivery of cells into the ischemic area. The injection can be made during open heart surgeries such as myocardial revascularization or through mini-thoracotomies, a less invasive approach indicated for patients with HF or reduced functional capacity^[7]^.

More recently, scaffold-based systems using biological or synthetic tridimensional structures had emerged as a promising tool for delivering SC into the myocardium, allowing SC to proliferate and differentiate. For this purpose, a great potential was found on the human amniotic membrane (hAM).

The hAM was first evaluated as a possible biomaterial in 1910, when John Davis used fresh membranes to the treatment of skin disorders. Since then, other medical specialties such as ophthalmology, urology, orthopedics, and gynecology had explored its use. The most attractive factor in using hAM as a biomaterial is related to its intrinsic characteristics of biocompatibility, because it does not cause an immunological reaction in the host and is also easily obtained and processed, not generating significant costs for its acquisition.

The amniotic fluid may be a possible source of SC, and differentiation into cardiomyocytes is a possible mechanism of action. However, similarly as the SC, this hypothesis is still controversial, and the most recent studies had been focused on the reduction of inflammatory response, prevention of cell death, and angiogenic potentials, mainly related to the hAM anti-inflammatory and anti-fibrotic effects^[8]^.

The hAM can be injected as a hydrogel or as a patch in the infarcted area. The patch implant may have a benefit over the hydrogel implant due to its three-dimensional support, preserving the hAM mechanical strength, which attracts and stimulates the survival of neighboring cells. Also, this three-dimensional structure-property can serve as a medium of cell culture, proliferation, and differentiation, even assisting as a method of cell delivery to the myocardium. Recently, we had used hAM as a scaffold for delivering anti-inflammatory nanoparticles in a rat model of HF, and the results after 30 days showed functional improvements and ventricular antiremodeling effects^[9]^.

Other tissues from multiple biological sources have been evaluated as possible scaffolds for SC delivery and cell repair in the setting of HF. Natural collagen and fibrin polymers were initially used, but the major limitation was related to their fast degradation and low mechanical strength.

The porcine intestinal submucosa was an alternative for these limitations, and its use has been explored as a patch for urinary tract reconstructions and vascular grafts, demonstrating superior results when compared to other collagen-composed materials, due to its three-dimensional architecture, rich vascularization, and the presence on its surface of growth and pro-angiogenic factors, collagen, laminin, and glycosaminoglycans. These natural components are able to promote the interaction of the host cells and the implanted material, allowing multiple cell adhesion and integration^[10]^. Despite this, an important factor to be considered is related to its possible immunogenic factor, for being a xenogeneic graft. Nevertheless, its potential use for tissue repair and regeneration cannot be discarded and future studies should focus on evaluating this scaffold as a possible form of delivery of SC to the myocardium, similarly to hAM.

Lastly, hydrogels-based approaches for tissue engineering are also a promising alternative for cardiac regeneration in models of HF. The hydrogels can be obtained from multiple sources such as collagen, proteoglycans, synthetic polymers, and from specific cardiac compartments^[11]^. The application form is mainly related to direct delivery to the myocardium, stimulating angiogenesis, and providing cell growth factors.

In conclusion, the most important factor to be considered when using a cell-based approach to the treatment of HF is related to its etiology and the underlying pathologies, defining whether it is fibrotic, ischemic, idiopathic, and/or inflammatory dilated myocardiopathy. Based on these considerations, specific treatments with the characteristics of each graft alone or together should be proposed.
